# How well must climate models agree with observations?

**DOI:** 10.1098/rsta.2014.0164

**Published:** 2015-10-13

**Authors:** Dirk Notz

**Affiliations:** Max Planck Institute for Meteorology, Bundesstrasse 53, 20146 Hamburg, Germany

**Keywords:** Earth System Models, sea ice, evaluation

## Abstract

The usefulness of a climate-model simulation cannot be inferred solely from its degree of agreement with observations. Instead, one has to consider additional factors such as internal variability, the tuning of the model, observational uncertainty, the temporal change in dominant processes or the uncertainty in the forcing. In any model-evaluation study, the impact of these limiting factors on the suitability of specific metrics must hence be examined. This can only meaningfully be done relative to a given purpose for using a model. I here generally discuss these points and substantiate their impact on model evaluation using the example of sea ice. For this example, I find that many standard metrics such as sea-ice area or volume only permit limited inferences about the shortcomings of individual models.

## 1. Introduction

Climate models are tools that have been developed to understand and to predict specific features of the real climate system of our Earth. In order to be useful for this purpose, it is necessary to evaluate the capability of the models to realistically represent these features. Such model evaluation is most commonly based on the direct comparison between simulation results and measurements of individual observables. Related studies often imply that such a comparison can result in absolute statements regarding model quality, which is reflected, for example, in rankings of model simulations based on their agreement with a specific observable, in the misleading use of terms such as ‘verification’ or ‘validation’ [1], or in generic calls that models need to be improved that fail to match a specific observable.

However, such assessments ignore the fact that models are tools that are in practice used to answer specific questions, be it to project the global temperature at the end of this century or to understand how a melting of the Greenland ice sheet affects the global ocean circulation. As climate models are only tools, they do not have a generic quality that one could usefully evaluate independent of the specific purpose for which a given model is being used. This line of thinking has been very clearly laid out by Parker [2] who argues that the significant simplifications underlying any climate model imply ‘that the complex hypothesis embodied by a climate model is false’, which is why only a model's ‘adequacy for a specific purpose’ can be evaluated.

In this contribution, I examine such evaluation of adequacy, or usefulness, in more detail. In doing so, I establish three points: first, the usefulness of a climate-model simulation cannot be inferred solely from its degree of agreement with observations. Second, the suitability of a metric for model evaluation depends crucially on the given research question. And third, model selection based on model evaluation can be counter-productive if one aims at robustly reducing the uncertainty range of future projections.

To establish these points, I here use the example of sea ice. This focus, however, does not limit the general validity of this analysis. It simply allows this contribution to remain in line with the other contributions in this issue. Furthermore, the example of sea ice makes it straightforward to concretize the main findings of this paper.

Take, for example, the magnitude of the loss of Arctic sea ice. One might think that any major mismatch between the observed loss and the loss simulated by a model necessarily implies that the model does not capture the relevant physics of sea-ice loss and needs to be improved. However, as discussed in more detail below, any such mismatch might simply be caused by the chaotic nature of the climate system, which would largely preclude inferences about the usefulness of the model for long-term projections based on this metric. A helpful analogy might be the case of a numerical model of casting a die that produces in three simulations a 1, a 3 and another 3. These simulation results do not allow one to infer that the model needs to be improved even if casting a real die results in a 6.

Internal variability is only the most prominent factor that often hinders climate-model evaluation, but there are others. For example, the tuning of individual models to match specific metrics can cause a false sense of adequacy of the model for a particular purpose. If, say, a model is tuned to match the observed sea-ice area over the past 10 years, this does not imply that the model's physics allows the model to give credible projections of the future evolution of the sea-ice cover. Other factors that hinder a direct evaluation of adequacy relate to the possible uncertainty of the forcing used in a model, or to the difficulties in establishing a link between the good modelling of the past evolution of some observable and an equally good modelling of the future evolution of that observable.

These factors always affect model evaluation, but their impact is very different for different research questions. For example, while a mismatch between modelled and observed sea-ice area might be irrelevant if one examines whether a model can simulate the long-term evolution of the climate, such a mismatch is very relevant if one tries to determine whether a model can adequately simulate the short-term evolution of sea ice: a model that simulates far too much sea ice in the Arctic for a particular June is unlikely to be adequate for estimating whether the Northern Sea Route will become navigable in the following July. This illustrates that in any model-evaluation study, the usefulness of a particular metric first needs to be demonstrated for the particular purpose for which a simulation is supposed to be used.

In the following, I exemplify these points through a case study that aims at estimating when the Arctic Ocean might become virtually free of sea ice during summer for a given emission scenario. As discussed in more detail in §3, the full CMIP5 model ensemble gives a very wide range as to when this might happen. A standard approach for narrowing down this range is based on evaluating individual models through a direct comparison with observations. The models that are thus shown to be inadequate for simulating the past evolution of the sea-ice cover are considered as inadequate for simulating the future evolution of the sea-ice cover, and hence excluded from the ensemble. This then results in a smaller ensemble with a smaller uncertainty range. However, this approach carries with it some severe difficulties, which are outlined in §4. In particular, the impact of internal variability, observational uncertainty, the unclear relevance of a metric, the tuning of the models and the unclear link between the past and the future evolution of the system are analysed. In §5, these results are briefly discussed. Building on this discussion, §6 briefly describes some possible ways forward. The paper concludes with a summary of the main findings.

## 2. Material and methods

I analyse the period 1975–2005 of the historical simulations from the CMIP5 archive that describe the past sea-ice evolution resulting from the observed evolution of, for example, solar variability, greenhouse-gas levels and volcanic eruptions, and the period 2005–2100 of the RCP simulations for the possible future evolution of the sea ice resulting from the Representative Concentration Pathways as described by the CMIP5 protocol [3]. Simulations from 28 different models are used, providing a total of 107 historical simulations, 42 simulations for the RCP 2.6 scenario, 54 simulations for the RCP 4.5 scenario and 57 simulations for the RCP 8.5 scenario. For each scenario, the number indicates the amount of anthropogenically caused radiative forcing from well-mixed greenhouse gases in units of watts per square metre by the end of the twenty-first century.

For all simulations, I calculate monthly mean sea-ice area and monthly mean sea-ice volume in the Arctic. Sea-ice area is calculated by multiplying the area of the model grid cells with their fractional sea-ice cover as provided by the CMIP5 archive, and then summing over all grid cells of the Northern Hemisphere. Sea-ice volume is calculated by multiplying the area of the model grid cells with their sea-ice volume per grid-cell area, which is the ‘equivalent sea-ice thickness’ that is provided by the CMIP5 archive. For both measures, linear trends are calculated as a least-square fit to the resulting timeseries.

As an estimate of true sea-ice coverage, I use sea-ice area as derived from satellite retrievals of passive microwave emissions. As different algorithms result in different estimates of sea-ice concentration, I here use two different algorithms, namely the Bootstrap algorithm [4] and the NASA Team algorithm [5]. Both algorithms cover the period from 1979 until today. I use sea-ice area as the main metric of sea-ice coverage to avoid some of the undesirable effects that result from the more commonly used nonlinear metric of sea-ice extent [6], noting that the use of two algorithms that are both based on passive-microwave emissions only provides a lower limit of observational uncertainty.

Because there is no similarly long observational timeseries of sea-ice thickness, I use for an estimate of the true sea-ice volume simulations of the Pan-Arctic Ice-Ocean Modelling and Assimilation System PIOMAS [7]. This system consists of an ocean–sea-ice model that is driven by NCEP/NCAR atmospheric reanalysis data, with an additional assimilation of satellite-retrieved sea-ice concentration. For the past decade, simulations of sea-ice thickness from PIOMAS agree well with satellite estimates [8], though the reliability of these estimates is sometimes not clear [9]. For earlier decades, sporadic comparisons of PIOMAS simulations with point observations also suggest a reasonable agreement of PIOMAS simulations with reality, though the uncertainty there is obviously high because of the low spatial and temporal coverage of the observations.

Section 4 describes how these data sources are used to possibly reject individual models for the case study.

## 3. Estimating the timing of an ice-free Arctic Ocean

**Figure 1.**
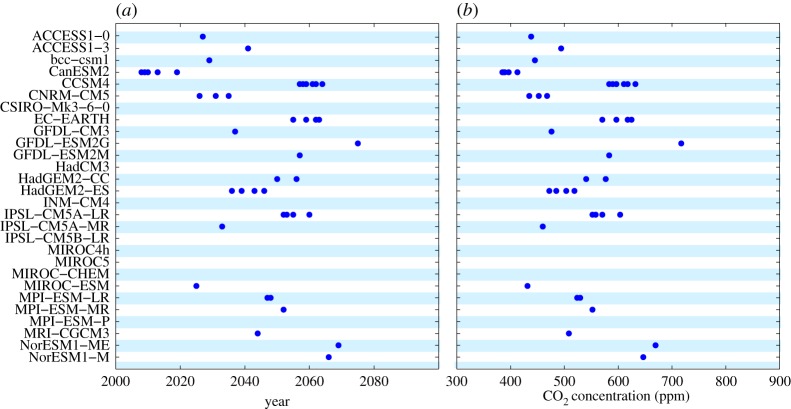
(*a*) Each dot specifies for a particular simulation the first year during which Arctic September sea-ice area drops below 1 million km^2^ for CMIP5 simulations under the scenario RCP 8.5. The respective model is identified along the *y*-axis. (*b*) Each dot specifies the CO_2_ concentration at which Arctic summer sea-ice area drops below 1 million km ^2^ for the RCP 8.5 scenario. No dot indicates that the specific model only gets ice-free after the year 2100 and a CO_2_ concentration of more than 900 ppm.

**Figure 2.**
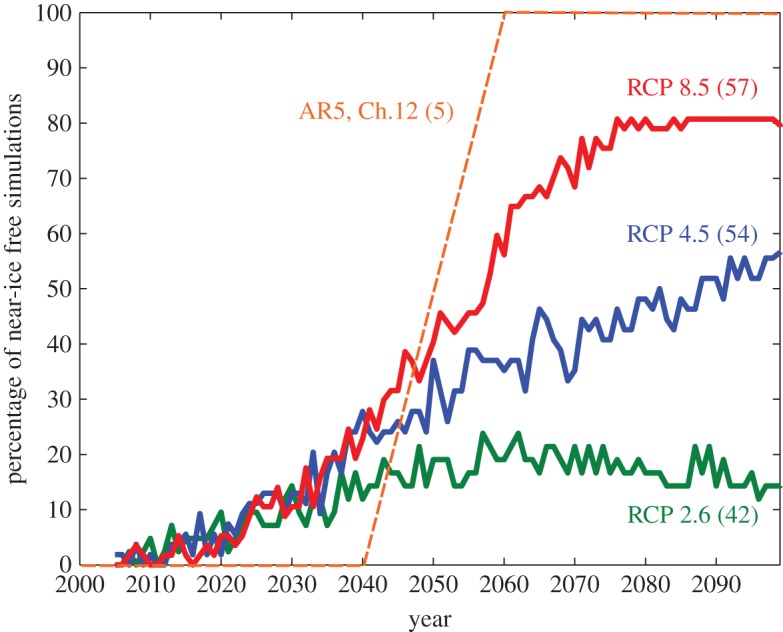
Percentage of CMIP5 model simulations that have a September Arctic sea-ice cover below 1 million km^2^ for any particular year. For each scenario, the number in brackets indicates the number of simulations that were available in the CMIP5 archive. The orange dashed line represents the range of 2040–2060 during which the Arctic becomes ice-free in five selected models [10].

The time and/or the atmospheric CO_2_ concentration at which the Arctic Ocean is virtually free of sea ice in summer has received considerable public attention in recent years. I here define that time as the first year in which September sea-ice area drops below 1 million km^2^, noting that the terms September and summer are used synonymously in this contribution. The CMIP5 simulations that are analysed here differ substantially in their related projections, with a temporal range for the first year of a virtually ice-free Arctic spanning in the RCP 8.5 scenario from 2005 until not within this century (figure 1*a*). Assuming initially that all these simulations are equally likely to be correct, one can determine a distribution of the percentage of models that simulate an ice-free Arctic during summer in a particular year (figure 2, red, green and blue lines). Given the breadth of the resulting distribution, it can be desirable to narrow down the distribution by excluding individual simulations from the ensemble. Such a narrowing down of the projection range might in particular be desirable from a policy perspective, as it reduces the uncertainty of scientific information on which a specific policy can eventually be based. This might be one reason for why such a reduced ensemble is discussed, for example, in ch. 12 of the 5th IPCC report [10], represented roughly by the dashed orange line in figure 2.

It is generally not obvious that a meaningful rejection of individual simulations based on the information we have available today should be possible, because the simulations describe the evolution of a dynamic system that is in part determined by chaotic fluctuations. A mismatch between a simulation and an observation hence does not necessarily imply that that simulation is unphysical. In our case, however, rejection of individual models should be possible in principle, as individual models that contributed several simulations to the CMIP5 archive typically result in a range of around 20 years as to the timing of a largely ice-free Arctic Ocean. This range represents the modelled uncertainty of the timing of an ice-free stage that is caused by chaotic internal variability. If we assume that a range of around 20 years uncertainty holds for all models, then for the period 2020 to 2080 only about six to eight models lie within this uncertainty range for any eventually observed year of an ice-free Arctic. The other approximately 20 models are then, for whatever reason, clearly inadequate for estimating the timing of an ice-free Arctic Ocean, as all their projections are more than 20 years away from the observed time. This finding even remains correct if the true internal variability of the climate system is larger than that given by the models: in that case, the models are inadequate simply because they underestimate the true internal variability. Hence, it seems like a promising endeavour to try to reject individual model simulations based on the information we have at hand already now.

A similar analysis can be carried out for the CO_2_ concentration at which the models project below 1 million km^2^ of ice coverage in the Arctic Ocean. Such an analysis gives a range from below 400 ppm to around 1100 ppm atmospheric CO_2_ concentration (figure 1*b*). With a spread of around 100 ppm for individual models, it will again be possible to determine that many of these models are inadequate to determine the threshold CO_2_ levels for an ice-free Arctic Ocean once that state has been reached. For a robust assessment of this inadequacy, it might be necessary to rerun models with the actually observed forcing, as the CO_2_ level of an ice-free Arctic depends for individual models on concentration pathways. Most models reach in their RCP 4.5 scenarios eventually an ice-free stage at CO_2_ concentrations at which the Arctic is still ice covered in the RCP 8.5 scenarios. This implies that there is some delay for the sea-ice response to changes in CO_2_ concentration [11]. Nevertheless, in principle it should be possible to reject many of these models for the purpose of determining the CO_2_ levels at which the Arctic becomes ice-free.

In the following, I will discuss why such a rejection of individual model simulations is often very difficult in practice, even if we have just established that it should be possible in principle. In doing so, I will not describe all possible ways that one could try to reject individual models but simply use a few specific metrics to exemplify some generic difficulties of this approach. Later, in §6, I will then outline possible ways to overcome these difficulties.

## 4. Obstacles for estimating the adequacy of a simulation for a particular purpose

### (a) Internal variability

**Figure 3.**
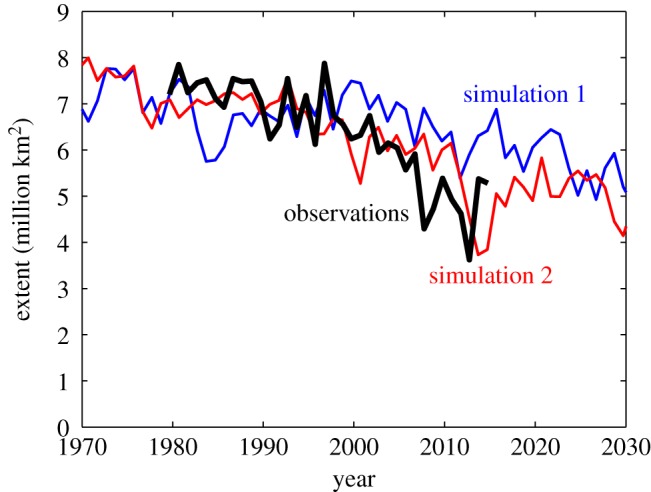
Comparison of satellite-retrieved and simulated evolution of September Arctic sea-ice extent. The two simulations were both carried out with the Max Planck Institute for Meteorology Earth System Model MPI-ESM and differ only very slightly in their initial condition at the beginning of the historical simulation in 1850. From 2005 onwards, the simulations follow the RCP 4.5 scenario.

Arguably, the most common obstacle that one faces when trying to reject a climate-model simulation as inadequate for a specific purpose derives from the internal variability of the climate system. Such variability is caused by the inherently chaotic nature of the atmospheric and oceanic circulation, which, for example, prohibits weather forecasts beyond about two weeks. Long-term climate projections can therefore only ever give a possible range for the temporal evolution of any specific variable, and the same model can result for the same external forcing in a multitude of possible trajectories for the time evolution of any climate metric. Take, for example, the three ensemble members of the Max Planck Institute Earth System Model MPI-ESM in its low-resolution set-up MPI-ESM-LR that were submitted to the CMIP5 archive [12]: for the same model and the same external forcing, one of these simulations projected an increase in Arctic sea ice over the period 1979–2012, while another simulation resulted in a decrease of Arctic sea ice similar to the one observed (figure 3). The fact that one simulation resulted in an increase in Arctic sea ice does not tell us anything about the capability of this model to realistically capture the processes that determine the future evolution of Arctic sea ice: despite a stark disagreement between this simulation and the observed sea-ice evolution, the model might well be very useful for our purposes, as reflected by the fact that another simulation with the same models matches the available observations quite well. This is simply a reflection of the fact that reality just realized a single trajectory of the infinite number of possible trajectories that are allowed for any given external forcing to the system.

The simple example depicted in figure 3 indicates how important it is to reasonably take internal variability into account for any attempt to reject a specific model for any purpose that is affected by internal variability. This hence usually affects all applications of climate model simulations that span time scales on which internal variability of the specific observable becomes a dominant source of fluctuations. It is often assumed that a period of 30 years is sufficient to neglect internal variability of the Earth's climate system, but, as also shown in the following, this assumption usually does not hold during periods of a rapidly changing climate [13].

A major challenge for taking internal variability into account during model evaluation lies in the difficulty of estimating the true internal variability based on the shortness of the available observational record. We have a consistent timeseries of sea-ice area retrievals from satellite from 1979 onwards and have no comparable timeseries for sea-ice volume at all. Previous studies have therefore estimated internal variability either from the fluctuation of the observed timeseries around the long-term trend [14] or by considering the spread of multiple simulations from individual models [6,15] that was assumed to be valid also for those models that only provided a single simulation to the timeseries. None of these methods, however, considers the strong and random temporal variability of many sea-ice-related parameters. Doing so allows one to obtain a more realistic estimate of internal variability, as considering temporal variability can be interpreted as a synthetic extension of ensemble size. This then results in a much larger estimate of internal variability than that given in previous studies.

In order to estimate an internal variability from such a synthetically increased ensemble size, I examine 30-year trends from the CMIP5 archive that start between the simulated years 1975 and 1984. This results in 10 possible trends for each individual model simulation. I then assume that a plausible range for any observable is given by the range of simulation results from those models that capture the observed value in their range of simulations. This reasoning is based on the fact that we cannot exclude the possibility that the observed evolution of the climate system follows a rather unlikely extreme trajectory for the given change in forcing. Hence, for any observable those models that have the observed trajectory at the extreme upper end of their simulated trajectories must currently be assumed to be equally likely to be correct as those that have the observed trajectory at the extreme lower end of their simulated trajectories. Unless we obtain an independent estimate of internal variability, the range obtained is the possible range of climate trajectories. Hence, any individual simulation that falls into this range cannot *per se* be rejected as unrealistic.

One could argue that individual models can nevertheless be rejected, namely if the observed trajectory of the climate system does not fall into the model's ensemble spread. This reasoning would be correct if the ensemble spread of those models that submitted several simulations to the CMIP5 archive represented the entire range of internal variability of the individual models. This, however, does not seem to be the case. We have just started analysing a recent very large ensemble of 100 historical simulations with an updated model version MPI-ESM-1.1, finding that the spread of the simulated 30-year-long trends over the single period 1975–2004 is for this single model even larger than that given by all CMIP5 simulations from all models that were used to construct internal variability as described in the previous paragraph.

This indicates that the change in start dates can indeed primarily be interpreted as a synthetic increase of the ensemble size that we can analyse. The overall contribution to the range that stems from the fact that the examined variables do change over time independent of internal variability is very small: this forced component can be estimated by examining for each simulation a linear regression through the individual data points for all start years. Such an analysis shows that usually less than 10% of the total range can be explained by the change in forcing over the 10-year-long period. Such a small contribution does not qualitatively affect the present analysis, so it is neglected in the following for simplicity.

**Figure 4.**
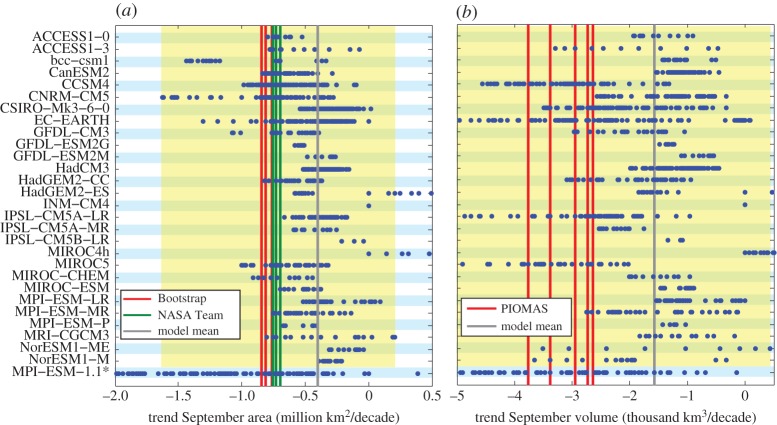
(*a*) Each dot represents for each simulation available in the CMIP5 archive the simulated trend in September Arctic sea-ice area for all 30-year-long periods starting between 1974 and 1984. The lines represent the respective values from satellite retrievals from 1979 onwards and for the multi-model mean across all simulations. The yellow area represents the range of trends consistent with the external forcing as given by those models that encompass the observed trend within their ensemble range. (*b*) Same as (*a*), but for September Arctic sea-ice volume. *The last row shows for reference the 30-year trends starting in 1975 for a 100-member ensemble with an updated version of MPI-ESM.

If we now apply this method to analyse the most often used metric, namely the trend in September sea-ice coverage, it becomes immediately clear that this measure is of very limited use if one tries to reject individual model simulations as unrealistic: the estimated internal variability is so large that it encompasses almost the entire range of simulated trends (figure 4*a*). Based on the present estimate of internal variability that is indicated by the yellow shading in the figure, the observed trend could have been twice as largely negative over the past three decades, or could even have been positive for the same external forcing. Accepting this range of internal variability, we can only directly reject those very few models as unrealistic that have ensemble members outside of the yellow shading in figure 4*a*.

Those models that have all their simulations inside the range of internal variability as estimated here could only be rejected if we could be sure that the model would not be able to simulate the observed trend within its ensemble spread even for a substantially increased ensemble size. However, as shown by the 100-member large ensemble simulation with MPI-ESM, it is very likely that for a very large ensemble, the observed trend of sea-ice area falls into the possible ensemble range of most models that we examine here. Hence, based on an analysis of the trend in sea-ice area, we cannot exclude any of them beyond those few that have simulations outside of the reasonable range.

The same holds for the trend in September sea-ice volume. Here, individual models simulate in their ensemble members trends that can range from a faster decline than given by the PIOMAS reanalysis to even an increase in sea-ice volume over the past 30 years (figure 4*b*). Hence, long-term trends in neither September sea-ice area nor September sea-ice volume allow us to reject individual models as inadequate for describing the response of the system to the change in forcing over the past 30 years. These observables are hence not useful measures if one aims to exclude individual models to estimate a narrower range of the point in time as to when the Arctic might become ice-free in summer. It should also be noted that any mismatch in these metrics between models and observations then obviously also does not allow us to robustly discredit the quality of CMIP simulations of the Arctic sea-ice evolution and to use this mismatch as the main argument to motivate sea-ice-related research.

### (b) Observational uncertainty

**Figure 5.**
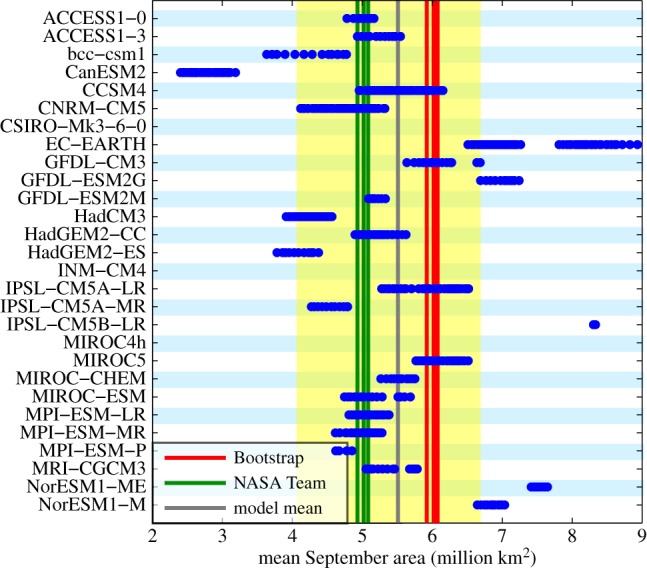
Each dot represents for each simulation available in the CMIP5 archive the simulated mean in September Arctic sea-ice area for all 30-year-long periods starting between 1974 and 1984. The lines represent the respective values from satellite retrievals from 1979 onwards and for the multi-model mean across all simulations. The yellow area represents the range of mean area consistent with the external forcing as derived from those models that encompass the observed trend within their ensemble range.

For other metrics, internal variability is just one contributor as to why one cannot reject individual models even if they do not agree with a specific observation. One example for this in the case of sea ice is given by the the satellite-based estimates of sea-ice area. For this observable, at least half of the plausible range for which models cannot be rejected stems from observational uncertainty (figure 5): mean sea-ice area as derived from the Bootstrap algorithm is more than 1 million km^2^ larger than that derived from the NASA Team algorithm. As we do not know which of these values is correct (and it is likely that indeed none of them is), I here take the spread of the observations as a direct measure for observational uncertainty and estimate the range of internal variability based on all those models that include either of the observational estimates within their ensemble spread. Doing so, I find that the internal variability gained such is amplified to a degree that only very few models clearly fall outside the uncertainty range of possible mean September sea-ice area over the past 30 years, which I estimate as spanning from 4 million km^2^ to just below 7 million km^2^ (figure 5).

As both satellite estimates are derived from the same underlying passive microwave data, the range of observational uncertainty might in reality be even larger than estimated here. Because of this large observational uncertainty of sea-ice area, model-evaluation studies often use the nonlinear metric of sea-ice extent, which is based on adding the size of all grid cells with at least 15% ice coverage. While sea-ice extent can more reliably be estimated from satellite than sea-ice area, its nonlinear construction causes a number of issues for model-evaluation purposes [6]. For example, the estimate of sea-ice extent depends crucially on the size of the underlying grid cells, with larger grid cells usually causing a larger estimate of sea-ice extent. Hence, usage of sea-ice extent is not necessarily a good solution to circumnavigate the observational uncertainty of sea-ice area in model-evaluation studies. Indeed, not even sea-ice area is an ideal choice, as it still allows for compensating errors in different regions. These could be considered by examining the RMS error of sea-ice concentration [6], for which again observational uncertainty is a major issue that hinders the robust evaluation of sea-ice coverage in our models.

### (c) Unclear relevance of a metric

**Figure 6.**
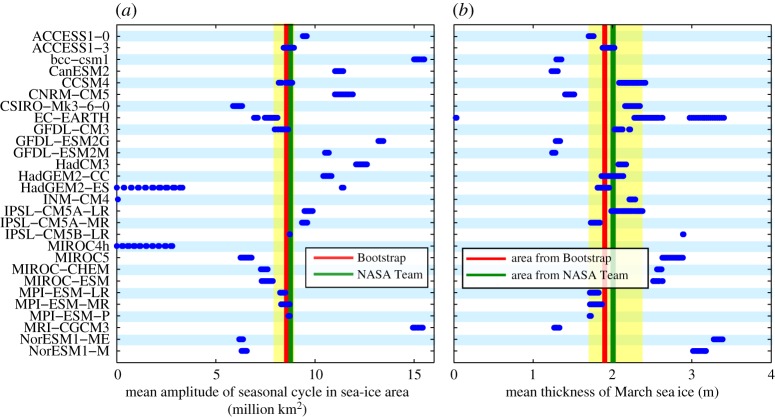
(*a*) Each dot represents for each simulation available in the CMIP5 archive the simulated mean amplitude of the annual cycle in Arctic sea-ice area for all 30-year-long periods starting between 1974 and 1984. The lines represent the respective values from satellite retrievals from 1979 onwards. The yellow area represents the range of mean amplitude consistent with the external forcing as derived from those models that encompass the observed trend within their ensemble range. (*b*) Same as (*a*), but for March Arctic sea-ice thickness. The observed values are derived by dividing PIOMAS estimates of sea-ice volume by satellite-derived sea-ice area from two different algorithms.

Based on the discussion so far, it is obvious that the ideal observable for model-evaluation purposes has little observational uncertainty, is barely influenced by internal variability and shows little spread for the various simulations of any particular model. Unfortunately, not all observables that have these properties are automatically useful for our purposes. This is primarily because often a causal link of the performance of the model for a particular metric and the timing of an ice-free Arctic is not clear. Take, for example, the mean seasonal cycle in sea-ice area (figure 6*a*), which is remarkably stable over the observational period: independent of the start date between 1979 and 1983, the 30-year mean amplitude lies at around 8.5 million km^2^. The observed standard deviation for any 30-year period during this time is only around 0.5 million km^2^. Also the model simulations have a comparably small range of the amplitude of the seasonal cycle across all ensemble members for 30-year periods starting between 1969 and 1989: the range of the amplitude lies typically at around 1 million km^2^. More importantly, however, only few simulations match the observed amplitude, even if one takes internal variability of around 1 million km^2^ into account. From the perspective of both improving our models and of narrowing down the uncertainty range for the future evolution of sea ice, this finding might initially appear promising: the seasonal cycle represents the model's response to a significant change in forcing between winter and summer, and as we have sufficient data available to reject individual simulations, this metric might be a good candidate for allowing us to improve projections of the future evolution of sea ice. Unfortunately, there is no clear correlation between the amplitude of the seasonal cycle in a particular model and the rapidity of the sea-ice loss in that model (not shown). In practice, it hence seems unlikely that an exclusion of models with an unrealistic seasonal cycle will provide for a much improved estimate of the timing of an ice-free Arctic.

### (d) Model tuning

In addition to such unclear relevance, also the tuning of individual models directly affects the usefulness of individual observables for model-evaluation purposes. Consider, for example, the mean sea-ice thickness during March, which is here calculated by dividing the March PIOMAS sea-ice volume by either the NASA Team- or the Bootstrap-derived sea-ice area. The reliability of this sea-ice thickness estimate hence rests on the reliability of both the PIOMAS model simulation and the uncertainty of sea-ice concentration retrievals, so the uncertainty of the ‘true’ value for this variable is obviously quite large. Notwithstanding these limitations that might eventually cause a slight shift in the true value of mean thickness of March sea ice, for our purposes it is most important to note that mean winter sea-ice thickness is a remarkably stable metric, with most models and the observations showing a spread of less than 50 cm (figure 6*b*). While a number of models clearly lie outside the range of observational uncertainty plus internal variability, it is not straightforward to show that these models are less useful to establish the timing of an ice-free Arctic Ocean than are the models that better agree with observations. This is in part because the linkage between mean winter sea-ice thickness and the timing of an ice-free Arctic Ocean is even less clear than a possible linkage with the amplitude of the seasonal cycle. However, even worse for our purposes, the mean thickness of winter sea ice is used in some of the models that we consider here as the main parameter that one attempts to match during the tuning of the model. Such tuning is necessary during the development of any climate model given the large number of processes that simply cannot be resolved explicitly and that hence need to be parametrized [16]. The models that use mean March sea-ice thickness for their tuning will almost automatically better agree with the observed state than models that use different parameters for the tuning. A fair assessment of the quality of the underlying model is hence not possible for this metric unless one carefully takes the tuning of every single model into account. It should be noted that in practice, most modelling groups will only use very few observational targets to tune their sea-ice model, which is why tuning becomes a less relevant factor in studies that consider many metrics simultaneously.

### (e) Unclear link between past and future

The discussion of individual metrics has so far been based on the tacit assumption that we can infer the quality of simulating the future evolution of a climate observable from the quality of simulating its past evolution for which observational data might be available. However, the link between the model performance for a past evolution and the performance for the future evolution of the system is often not clear. A trivial example for this fact is given in figure 3, where the obviously ‘poor’ simulation with an increase in sea-ice cover over the period 1979–2012 becomes almost identical to the much more ‘realistic’ simulation shortly after this period. This change in perceived quality is simply related to internal variability, but for the severe changes that the climate system of the Earth currently undergoes, additionally the relevance of individual physical processes is very likely to change with time. This implies that a model that did well in the past does not necessarily do well in the future and vice versa.

Sticking to the example of sea ice, the multi-year sea ice that used to be the prevalent ice type in the Arctic until some years ago has distinctly different properties compared with the first year ice that covers much of the Arctic Ocean today. If the processes that a particular model represents well are parametrized based on the properties of the multi-year sea ice, such a model is likely to suffer in performance when simulating the future evolution of the ice pack.

To establish a clear link between the past and the future evolution of a specific observable, one ideally uses observational data from periods where the climate state was similar to the one that one aims at simulating. This is reflected by the growing interest in paleo data in particular from those periods where the climate state of the Earth was distinctly different from what it is today. Observational data from such periods allow us to evaluate the model performance over a large range of climate states [17], which forms one of the so-called severe tests that are an important means to increase our trust in Earth System Model simulations [18].

Unfortunately, for many observables we currently have no reliable data that would allow us to test climate-model performance for different climate states. In these cases, the model simulations themselves allow one to estimate the linkage between the state of the system in a particular year and the state of the system some years later. This is discussed in more detail in §6.

## 5. Discussion

The past evolutions of sea-ice area and sea-ice volume are often taken to be the most relevant observables for a possible narrowing down of the temporal range for a possibly sea-ice-free Arctic Ocean, as they most directly relate to this observable. It is hence sobering that neither the long-term trend nor the long-term mean of either area or volume allow us to substantially reduce the uncertainty as to when the Arctic Ocean might become free of sea ice in summer based on the rejection of individual models from the ensemble. Not only is the link between the past and the future evolution of these observables often not clear, their temporal and spatial variability is additionally too large to allow us to reject a significant number of model simulations as unreasonable.

Such a large internal variability of an observable has sometimes counterintuitive implications regarding model quality. For example, it is usually not possible to use such an observable to draw firm conclusions regarding a possible improvement in model quality for CMIP5 simulations relative to CMIP3 simulations: the fact that, in our case, the CMIP5 ensemble more closely matches the observed trend and observed mean of sea-ice area and sea ice volume compared with CMIP3 simulations [14] could, at least in principle, also indicate an on average worse quality of our models: if the observed sea-ice evolution over the past decades forms an extremely unlikely realization for the given atmospheric forcing, one would expect the real evolution of the sea-ice cover to only show up in but very few simulations. It is hence by no means a desirable aim to have the real state of the climate system lie roughly in the center of any model ensemble, nor is it an indication of a particularly realistic model ensemble if this is achieved.

While the severe difficulties in narrowing down projection ranges through the rejection of individual models have here only been examined for sea ice, our findings are generally true for any metric for which the observational period is too short to robustly remove the impact of internal variability, where in our case even 30 years are too short a period to fulfil this criterion. The most prominent example for a metric with similar issues is arguably the evolution of global-mean surface temperature, where the 15-year-long hiatus that occurs in temperature records that do not include measurements in the Arctic does not allow us to robustly reject climate-model simulations as unrealistic [19].

It should also be noted that even if one manages to robustly reject a particular simulation of, say, Arctic sea-ice evolution as unrealistic, this does not necessarily imply that the sea-ice component of that particular model is insufficient. This is exemplified by the fact that the sea-ice simulations of the CMIP5 ensemble differ substantially from those of the CMIP3 ensemble, while the sea-ice model components of most models remained largely unchanged from CMIP5 to CMIP3. The change in simulated sea ice is hence only explicable through a different tuning of this component, or through changes in other components of the Earth System Models. Such changes will usually directly affect the sea-ice simulations as on the temporal scales typical for large-scale models the time evolution of the sea-ice cover depends primarily on the total amount of heat that is provided to the ice from both the atmosphere and the ocean. If this amount of heat is modelled unrealistically, not even the best sea-ice model could produce a reasonable time evolution of the sea-ice cover. By contrast, if the amount of heat that is provided to the ice is modelled realistically, even a relatively simple sea-ice model component will produce reasonable results regarding, for example, the time evolution of sea-ice volume. While a better understanding of sea-ice-related processes is hence clearly desirable from a scientific point of view, it is often not clear that such improved understanding will also lead to measurably improved simulations of sea ice in large-scale models. Also the approach sometimes taken to examine shortcomings of Earth System Model components by examining them in a stand-alone, uncoupled set-up has quite substantial limitations. Such set-ups automatically remove many of the feedbacks that govern the evolution of the system and hence also usually do not allow for a direct assessment of the quality of individual Earth System Model components.

## 6. Ways forward

In the previous sections, I have laid out why it is very difficult to narrow down the uncertainty range of the timing of near-complete Arctic sea-ice loss by rejecting individual models from the ensemble based on their agreement with observations. However, as described, for example, by Collins *et al.* [20], one can sometimes circumnavigate these difficulties by examining the entire model ensemble for emerging constraints that allow one to establish the most likely future evolution of the climate system based on its past evolution. This approach does not require one to exclude models from the ensemble, and indeed it is most robustly used by establishing relationships that hold for the greatest possible number of models.

In the case of sea ice, a number of studies have used this approach. They established, for example, a relationship between the trend of Arctic sea-ice coverage over the past 30 years and the amount of sea ice that remains by the mid-twenty-first century [20,21], or between the sea-ice area over the past few years and the sea-ice area in 10 years' time [22]. These relationships allow one to estimate the most likely future evolution of the Arctic sea ice cover by establishing the functional relationship between an observable from the past and the evolution of the same or some other quantity in the future and to then constrain the future from the actually observed evolution of the climate system. This method has the added advantage of also resulting in an estimate of uncertainty based on the robustness of the statistical relationship.

The approach of using emergent constraints is conceptually different from the approach of excluding models from the ensemble, in particular as it does not require any particular model to be close to the observed state. The usefulness of emergent constraints instead derives from the robustness of statistical (and ideally also physical) relationships between climate observables from many models, and actually profits from a rather large spread of contributing model simulations. Hence, for using this method it is neither necessary nor desirable that the models converge on the single trajectory that the real world followed, even though this is sometimes seen as the ultimate aim of climate-model development.

Such emergent constraints can allow for robust insights into the future evolution of the climate system even for variables that have large internal variability. They, however, do not judge the quality of individual models and are hence not useful if one aims at improving the physical realism of the models themselves. To achieve model improvement, model evaluation remains the ultimate pathway (in a sea ice context cf. [23–26]). Some of the observables that we discuss here can be useful to identify shortcomings of our models, as exemplified by the mean March sea-ice thickness or the seasonal cycle. However, a more promising route to model improvement is given by a process-based evaluation of model performance rather than sticking primarily to an evaluation that considers only individual observations. Such a process-based evaluation of model performance can initially be carried out entirely in the realm of models, by focusing not only on identifying the differences between individual models but also on understanding the underlying sources for these differences (cf. [27]). Possible processes that one could evaluate in a sea-ice context are the relationship between thickness and growth, the relationship between sea-ice concentration and cloudiness or the seasonal evolution of ice albedo, to name but a few. An improvement of our models based on an improved representation of these processes would pave the path for ever more reliable simulations of the climate system of our Earth—and might ultimately even allow us to further narrow down the spread as to when the Arctic becomes potentially sea-ice-free during summer.

## 7. Conclusion

In this contribution, I have used the example of Arctic sea ice to examine why the agreement between model simulations and observations only provides limited insight into the usefulness of a climate model. The main conclusions of this analysis are the following.

(i) Climate models can only meaningfully be evaluated relative to a specific purpose. For such an evaluation, the suitability of any given metric for that specific purpose needs to be demonstrated.
(ii) Any mismatch between observation and model simulation might simply be caused by chaotic internal variability. This variability hence must be considered if one wants to draw firm conclusions about the shortcomings of a model based on its lacking agreement with observations.
(iii) For any study that aims at narrowing down projection ranges, the link between the models' performance in simulating the past evolution of the climate system and their performance in simulating the future must be demonstrated.
(iv) As we cannot usually know if the observed evolution of the climate state follows a rather unlikely pathway for a given forcing, it is not necessarily a desirable aim to have observations lie in the centre of a model ensemble.
(v) A metric that was used as an aim for tuning during the development of individual models will often give irrelevant results regarding the adequacy of models for a particular purpose.
(vi) Because of internal variability, those metrics usually allow for most robust insights during model evaluation that are based on probability distributions over many data points, rather than on long-term means or long-term trends.
(vii) A 30-year-long averaging period can be insufficient to substantially reduce the impact of internal variability, in particular during periods of a significant change in external climate forcing.
(viii) Statistical relationships of emerging constraints can give reliable insights into the future evolution of the climate system. These insights are the more reliable the larger the variety of underlying models is.

Regarding the specific case of sea ice that was examined here, the following concrete findings have been made.

(i) It is difficult to robustly narrow down the uncertainty range as to when the Arctic becomes free of sea ice during summer by rejecting individual models from the ensemble. This is primarily because the link between the past and the future evolution of the modelled sea-ice state is often not robust enough, and because internal variability hinders us to reject most models as unrealistic. Hence, in addition to estimates from emerging constraints, a probability distribution of an ice-free Arctic Ocean as estimated from all models might be the most insightful way of informing policy makers (figure 2).
(ii) Both the simulated mean and the simulated trend of sea-ice area and sea-ice volume are too variable even for 30-year-long periods to robustly ascribe a possible mismatch between models and observations to a shortcoming of most models. This is partly related to the fact that we lack an independent estimate of the true internal variability of the system.
(iii) The usefulness of the long-term mean or the long-term trend of the 35-year-long record of Arctic sea-ice observations is limited by the fact that its length only allows for five data points for any 30-year mean value or any 30-year trend. These five data points are too variable in time and too much influenced by internal variability to allow for a robust model evaluation. By contrast, a 35-year-long record provides 35 data points for, say, the seasonal cycle, which then allows for a much more robust assessment of the adequacy of individual models.

In summary, despite its widespread use, the direct comparison of model simulations with observations often only allows for rather limited inferences about the shortcomings of a particular climate model. Such shortcomings can, by contrast, be directly inferred from process-based evaluation studies, which can hence more robustly guide the development of ever better tools for understanding the climate system of our planet.
